# Lisosan G Protects the Retina from Neurovascular Damage in Experimental Diabetic Retinopathy

**DOI:** 10.3390/nu10121932

**Published:** 2018-12-05

**Authors:** Rosario Amato, Maria Grazia Rossino, Maurizio Cammalleri, Filippo Locri, Laura Pucci, Massimo Dal Monte, Giovanni Casini

**Affiliations:** 1 Department of Biology, University of Pisa, via San Zeno 31, 56127 Pisa, Italy; rsr.amato@gmail.com (R.A.); rossinomariagrazia1@gmail.com (M.G.R.); maurizio.cammalleri@unipi.it (M.C.); filippo.locri@student.unisi.it (F.L.); 2 Interdepartmental Research Center Nutrafood “Nutraceuticals and Food for Health”, University of Pisa, via del Borghetto 80, 56124 Pisa, Italy; 3 National Research Council, Institute of Agricultural Biology and Biotechnology (IBBA), Pisa Unit, Via Moruzzi 1, 56124 Pisa, Italy; pucci@ibba.cnr.it

**Keywords:** neuroprotection, nutraceuticals, oxidative stress, retinal explant, streptozotocin, VEGF

## Abstract

Lisosan G (LG), a fermented powder obtained from whole grains, is a recognized antioxidant compound that improves the bioactivity and survival of different cell types. The purpose of this study was to investigate whether LG ameliorates both the neural and the vascular damage characterizing early stages of diabetic retinopathy (DR). The effects of LG were studied in cultured explants of mouse retinas challenged with oxidative stress (OS) or in retinas of streptozotocin (STZ)-treated rats. Apoptosis, vascular endothelial growth factor (VEGF) expression, OS markers, blood-retinal barrier (BRB) integrity, and inflammation were assessed, while retinal function was evaluated with electroretinogram (ERG). LG extensively inhibited apoptosis, VEGF expression, and OS both in retinal explants and in STZ rats. In addition, STZ rats treated with LG displayed an almost total BRB integrity, reduced levels of inflammatory markers and a partially restored visual function as evaluated with ERG. In summary, we demonstrated that LG exhibits antioxidant and anti-inflammatory effects that exert powerful protective actions against neural and vascular defects characteristic of DR. Therefore, LG-containing foods or supplements may be considered to implement DR treatments.

## 1. Introduction

Diabetic retinopathy (DR) is a leading cause of visual impairment in both industrialized and developing countries. Although the pathology is currently treated with some success using intraocular administrations of drugs inhibiting abnormal proliferation of retinal vessels, alternative and possibly less invasive methods of intervention are urgently needed.

A growing body of experimental evidence indicates that, in early phases of the disease, neuronal stress and neurodegeneration are important events that may cause vascular endothelial growth factor (VEGF) release and the development of microvascular lesions, indicating neuroprotection as a target of primary importance for DR treatment [1,2,3]. Neuronal damage in DR may be caused by a variety of chemical stressors including advanced glycation end-products, glutamate-induced excitotoxicity, altered glucose metabolism, and altered mitochondrial activity [1,3]. The formation of reactive oxygen species and consequent oxidative stress (OS) represent a sort of common etiopathogenic trigger induced by these stressors, ultimately causing cell death.

There is evidence that dietary intake of individual foods, macro or micronutrients, dietary supplements, and dietary patterns may have an impact on DR [4]. Therefore, a potentially rewarding strategy is that of searching for natural substances with recognized antioxidant power that can be easily administered with the diet. Indeed, different foods or dietary supplements may have therapeutic potential for DR treatment, however, bioavailability and the capacity of crossing the blood-retinal barrier (BRB) may represent serious problems in many cases [5].

Lisosan G (LG) is a fermented powder obtained from organic whole grains (*Triticum aestivum*), registered with the Italian Ministry of Health as a nutritional supplement. It contains the typical components of cereals and with the fermentation, it is enriched in bioactive substances such as phenolic components and alpha-lipoic acid [6]. It is also rich in flavonoids and flavonols, tocopherols and polyunsaturated fatty acids [7]. A molecular characterization of the components of LG has been provided previously [8], while a more specific analysis of the phenolic compounds contained in LG has been published recently [9]. Both in vitro and in vivo studies have shown that LG is highly effective in protecting and improving the bioactivity of different cell types, from hepatocytes to microvascular endothelial cells, through the control of both oxidative and inflammatory processes [6,7,8,9,10,11,12]. In particular, the positive effects of LG are likely to be associated with radical scavenging, attenuation of OS, strengthening of antioxidant defenses, and reduced nuclear translocation of nuclear factor kappa-light-chain-enhancer of activated B cells (NF-kB) [6,12], which is an oxidant-sensitive transcription factor responsible for regulating gene expression of factors involved in inflammatory responses. The antioxidant capacity of LG has been evaluated in vitro employing the oxygen radical absorbance capacity, the 2,2-diphenyl-1-picrylhydrazyl, and the cellular antioxidant activity tests/methods [9,13].

Since the antioxidant effects of LG have not been tested previously in the nervous system, in the present work we first explored the possibility that LG, by reducing OS, may decrease both apoptosis and VEGF expression in a mouse ex vivo retinal model that we have set up and used in recent works [2,14]. The results of these experiments were propaedeutic to a longer-term investigation in which we evaluated whether oral administrations of LG could counteract both the neural and the vascular damage typical of DR using a rat model of streptozotocin (STZ)-induced diabetes.

## 2. Materials and Methods

### 2.1. Reagents

LG was supplied by Agrisan Company (Larciano, Pistoia, Italy). Millicell-CM culture inserts were from Merck Millipore (Darmstadt, Germany). RNA isolation kit (RNeasy Mini Kit) and QuantiTect Reverse Transcription Kit were from Qiagen (Hilden, Germany). The master mix (SsoAdvanced Universal SYBR Green Supermix), the polyvinylidene difluoride membranes, and the enhanced chemiluminescence reagent were from Bio-Rad Laboratories (Hercules, CA, USA). Micro BCA™ Protein Assay Kit and primers were obtained from ThermoFisher (Waltham, MA, USA). The RIPA Lysis buffer, the rabbit polyclonal antibodies directed to the p65 subunit of NF-kB (NF-kB p65; sc-372), to NF-kB p65 phosphorylated at Ser 276 (pNF-kB; sc-101749), and to nuclear factor erythroid 2-related factor 2 (Nrf2; sc-13032) as well as the horseradish peroxidase-labeled mouse anti-rabbit secondary antibodies were purchased from Santa Cruz Biotechnologies (Santa Cruz, CA, USA). The rabbit polyclonal antibodies directed to VEGF receptor 2 (VEGFR2; ab39256) and to the VEGFR2 phosphorylated at Tyr 1054 and Tyr 1059 (pVEGFR2; ab5473) were purchased from Abcam (Cambridge, UK). The rabbit monoclonal antibody directed to cleaved caspase-3 (#9664) was purchased from Cell Signaling Technology (Danvers, MA, USA). The rabbit polyclonal antibody directed to cleaved caspase-3 (C8487) was purchased from Sigma-Aldrich (St. Louis, MO, USA). The Alexa fluor 546- and Alexa fluor 488-labeled goat anti-rabbit secondary antibodies were purchased from Life Technologies (Carlsbad, CA, USA). All other chemicals were purchased from Sigma-Aldrich.

### 2.2. Animals

The procedures were approved by the Commission for Animal Wellbeing of the University of Pisa (permission number: 0034612/2017) and were in compliance with the ARVO Statement for the Use of Animals in Ophthalmic and Vision Research, the Italian guidelines for animal care (DL 26/14), and the EU Directive (2010/63/EU). All experimental procedures were performed to reduce both animal suffering and the number of animals used. C57BL/6J mice and Wistar rats (Envigo, San Pietro al Natisone, Udine, Italy) were used in these studies. They were kept in a regulated environment (23 ± 1 °C, 50 ± 5% humidity) with a 12 h light/dark cycle (lights on at 8:00 a.m.) with food and water ad libitum.

### 2.3. Preparation of LG

LG is a powder obtained by fermenting and drying whole wheat flour from* Triticum aestivum* grains. The starter cultures typically consist of a mix of lactobacillus and natural yeast strains in a ratio of about 100:1 (Natural Sourdough). Once the product was fermented, it was dried using a vacuum pump at 20–25 °C temperature and 2 bar pressure until reaching 12% humidity (48–60 h for 100 kg material).

For the ex vivo treatments, LG was dissolved in distilled water. We found that the solubility limit of LG was 1 g LG in 15 mL water (that is 67 mg/mL), therefore we used this solution as a stock solution. Based on preliminary observations (Supplementary Figure S1), 1:10^4^ and 1:10^5^ dilutions were used in the experiments. The LG stock solution was sonicated and centrifuged for 10 min at 2300× *g* at 4 °C (Jouan CR3i centrifuge, Newport Pagnell, UK). The supernatant was collected, filtered (0.2 μm, VWR International PBI, Milan, Italy), and kept at 4 °C in the dark until use. For in vivo treatments, an LG water suspension of 0.11 g/mL, containing both the hydrophilic and the lipophilic components of LG, was used for oral administration by gavage.

### 2.4. Ex-Vivo Mouse Retinal Explants

Retinas from 4- to 5-week-old mice of both sexes were dissected in Modified Eagle Medium (MEM) and cut into 4 fragments. The fragments were transferred onto Millicell-CM culture inserts with ganglion cells up. The inserts were placed in 6-well tissue culture plates and exposed to OS using 1 mL of serum-free culture medium containing 100 µM H_2_O_2_ [14] and composed of 50% MEM/HEPES, 25% Hank’s buffer salt solution, 25% PBS, 25 U/mL penicillin, 25 mg/mL streptomycin, 1 µg/mL amphotericin B, and 200 µM L-glutamine. Control explants were incubated in the same medium without H_2_O_2_. LG was added to the culture medium at two different concentrations (0.67 or 6.7 µg/mL). The explants were incubated up to 5 days at 37 °C under a humidified 95%/5% (vol/vol) mixture of air and CO_2_. The medium was changed every other day.

### 2.5. In Vivo Rat Experimental Diabetes and LG Administration

Ten rats weighing 180–200 g received a single intraperitoneal injection of 65 mg/kg STZ dissolved in citrate buffer, pH 4.5. Five age-matched rats received an equivalent volume of the citrate buffer solution (from now on referred to as control group). Blood glucose was measured three days after the injection by tail sampling using a OneTouch Ultra glucometer (LifeScan Inc., Milpitas, CA, USA). The ten rats treated with STZ showed a stable glycaemia > 250 mg/dL and they were assigned to the diabetic group. Non-fasting glycaemia and body weight were monitored once a week until sacrifice. A week after diabetes onset, 5 rats from the diabetic group were randomly assigned to the LG treatment group and daily treated with about 0.9 mL of LG solution, corresponding to a dose of 0.5 g/kg LG, by oral gavage [8]. Five weeks after diabetes onset, the rats were sacrificed by intraperitoneal administration of a lethal dose of sodium pentobarbital. 

### 2.6. Quantitative Real-Time PCR

In all the experiments, three independent samples from each experimental group were analyzed. For the ex vivo explants, each sample was represented by 8 fragments from different retinas pooled together, while, for the STZ rat model, each retina represented a separate sample. Total RNA was extracted, purified and resuspended in RNase-free water using an RNeasy Mini Kit. After spectrophotometric quantification, 1 μg of total RNA was used to generate the first-strand cDNA using QuantiTect Reverse Transcription Kit. Quantitative real-time PCR (qPCR) was performed using SsoAdvanced Universal SYBR Green Supermix on a CFX Connect Real-Time PCR Detection System and software CFX manager (Bio-Rad Laboratories). qPCR primer sets were chosen to hybridize to unique regions of the appropriate gene sequence (Table 1). Amplification efficiency was close to 100% for each primer pair (Opticon Monitor 3 software; Bio-Rad Laboratories). Both mouse and rat target genes were concurrently assayed with Rpl13a, a constitutively expressed gene encoding for ribosomal protein L13A [15]. Samples were compared using the relative threshold cycle (Ct method). The increase or decrease (fold change) was determined relative to experimental control group after normalization to Rpl13a.

### 2.7. Western Blot

In all the experiments, three independent samples from each experimental group were analyzed. For the ex vivo explants, each sample was represented by 8 fragments from different retinas pooled together, while, for the STZ rat model, each retina represented a separate sample. Proteins were extracted using RIPA Lysis buffer supplemented with protease and phosphatase inhibitor cocktails. The amount of extracted protein was evaluated using Micro BCA™ Protein Assay Kit (Pierce Biotechnology, Rockford, IL, USA). An equal amount of proteins from each sample (30 μg) were subjected to SDS-PAGE and blotted onto polyvinylidene difluoride (PVDF) membranes (Bio-Rad Laboratories). Membranes were blocked for 2 h in 4%* w*/*v *Bovine serum albumin in TBST and incubated overnight with primary antibodies. The rabbit monoclonal antibody directed to active caspase-3 (1:500) and the polyclonal antibodies directed to NF-kB p65 (1:100), to pNF-kB (1:100), to VEGFR2 (1:400) and to pVEGFR2 (1:400) were used for membrane probing. After incubation with the appropriate horseradish-peroxidase-conjugated secondary antibody (1:5000), immunoreactive bands were visualized with the enhanced chemiluminescence reagent. Images were acquired using the Chemidoc XRS+ (Bio-Rad Laboratories). Quantitative optical density (OD) analysis of the bands was performed using Image Lab 3.0 software (Bio-Rad Laboratories). The data were normalized to β-actin, NF-kB p65, or VEGFR2, as appropriate.

### 2.8. Immunofluorescence

Mouse retinal fragments and eyecups from rat eyes were fixed in 4% paraformaldehyde in PBS and stored overnight at 4 °C in 25% sucrose in PBS. Subsequently, they were embedded in cryo-gel, frozen using liquid nitrogen, and cut into 10 μm thick coronal sections with a cryostat. The sections were incubated overnight with antibodies directed to active caspase-3 (rabbit polyclonal antibody C8487, 1:400 dilution) or to Nrf2 (1:50 dilution) and then with secondary antibodies conjugated with Alexa-Fluor-546 or Alexa-Fluor-488 at a dilution of 1:200 for 2 h at room temperature. Thereafter, the slides were coverslipped with Fluoroshield Mounting Medium containing DAPI (Abcam, Cambridge, UK) and images were acquired using an epifluorescence microscope (Nikon Europe, Amsterdam, The Netherlands). Quantification of caspase-3 immunostaining in the ex vivo model was performed by counting the number of immunopositive cells per section unit length from 4 different fragments for each experimental group. Nrf2 immunofluorescence was also investigated using a Zeiss Imager Z2 fluorescence microscope equipped with an ApoTome 2 system (Zeiss, Jena, Germany). In these images, the levels of Nrf2 immunofluorescence intensity were measured using the “Analysis” menu of Adobe Photoshop (Adobe Photoshop CS3; Adobe Systems, Mountain View, CA, USA). Briefly, Nrf2 immunofluorescence images were converted to grayscale and normalized to the background. Then, the area occupied by DAPI-stained nuclei was subtracted from the INL or the ONL and the mean grey value was measured in these non-nuclear regions. In the GCL, the mean grey value was measured within the region of DAPI-stained nuclei. For each retinal layer, 4 measurements were recorded in retinal sections deriving from different animals for each experimental condition.

### 2.9. Electroretinogram

Retinal function was examined once a week with scotopic full-field electroretinogram (ERG). After anesthesia, rat pupils were dilated with 0.5% atropine and saline solution drops were intermittently instilled to prevent ocular surface dryness and clouding. A heating pad was used to keep the body temperature at 38 °C. Two silver/silver chloride electrodes on the ocular surfaces and a forehead reference electrode allowed to record ERG responses from both eyes. A ground electrode was placed on the tail. ERG responses were evoked by a 1 log cd-s/m^2^ flash generated through a Ganzfeld stimulator (Biomedica Mangoni, Pisa, Italy). The amplitude of the a-wave was measured at a fixed time of 8 ms after stimulus onset to minimize contamination from non-photoreceptoral contributions [16]. The b-wave amplitude was measured from the trough of the a-wave to the peak of the b-wave. Mean amplitudes of a- and b-wave ERG responses were plotted. For each experimental condition, ERG analysis was performed on 3 rats.

### 2.10. Detection of Retinal Vascular Leakage by Evans Blue Dye

Rats were anesthetized with an intraperitoneal injection of 30 mg/kg sodium pentobarbital. Thereafter, the rats were perfused with Evans blue dye (0.5% in PBS) injected into the left cardiac ventricle. Then, the retinas were dissected, flat-mounted on microscope slides and examined with an epifluorescence microscope (Nikon Europe).

### 2.11. Statistical Analysis

Statistical significance was evaluated using one-way analysis of variance (ANOVA) followed by Newman–Keuls multiple comparison post-test. Despite the limited number of samples per group, a parametric analysis was performed as the data inside each sample were normally distributed. The results were expressed as mean ± SD of the indicated *n* values (Prism 5.03; GraphPad software, San Diego, CA, USA). Differences with *p* < 0.05 were considered significant. In respect of the 3Rs principles for ethical use of animals in scientific research, an a priori power analysis was conducted using the software G*Power 3.0.10 to determine the minimum number of animals necessary to obtain a statistical power of at least 0.80, with α = 0.05, in the presence of a large effect size as expected in these studies. After the data were collected, a post hoc power analysis was conducted in order to confirm that a reliable statistical power was obtained in the experiments. A power value of 0.80 was considered the required minimum value to reject the null hypothesis.

## 3. Results

### 3.1. Effects of LG Treatment on OS-Stressed Mouse Retinal Explants

**Figure 1:**
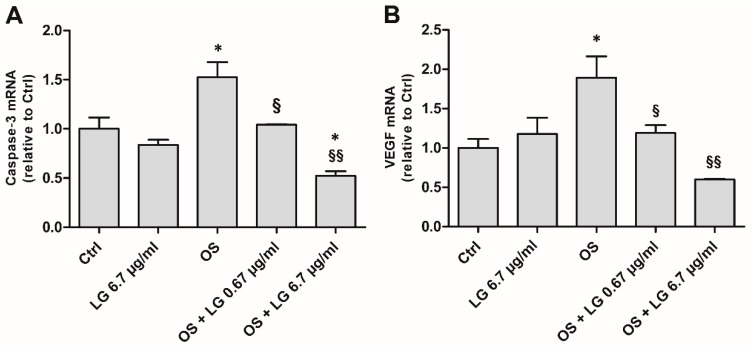
Caspase-3 (**A**) and vascular endothelial growth factor (VEGF), **B**) mRNA expressions evaluated with qPCR in ex vivo retinal explants cultured for 5 days in control (Ctrl) conditions, in the presence of 6.7 µg/mL Lisosan G (LG), or in oxidative stress (OS) with LG at 0.67 or 6.7 µg/mL. Each column represents mean ± SD. * *p* < 0.05 vs. Ctrl; ^§^ *p* < 0.05 vs. OS; ^§§^ *p* < 0.01 vs. OS; *n* = 3. Power value: 0.88 and 0.81, respectively.

As expected from our previous observations [14], after 5 days of incubation both caspase-3 (Figure 1A) and VEGF (Figure 1B) mRNAs were increased in OS, but the treatment of the explants with LG reversed the effects of OS in a dose-dependent manner. In particular, 0.67 µg/mL LG resulted in recovery of both VEGF and caspase-3 mRNA to control levels, while 6.7 µg/mL LG significantly reduced the level of caspase-3 mRNA even below control values. Treatment with LG alone (6.7 µg/mL) did not induce any changes. Since LG at both 0.67 and 6.7 µg/mL resulted in significant recovery, in the following experiments with ex vivo retinal explants the lowest dose of LG, that is 0.67 µg/mL, was used.

**Figure 2:**
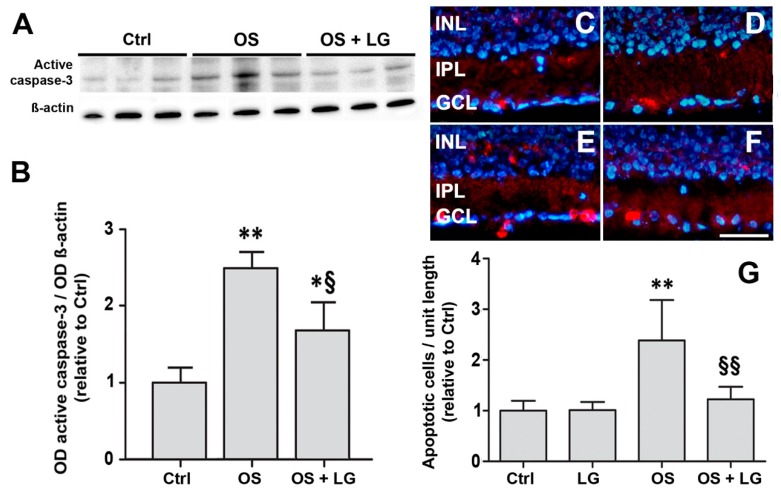
Ex vivo retinal explants cultured for 5 days. (**A**) Western blots showing immunoreactive bands of active caspase-3 and of ß-actin, used as an internal standard, in control explants and in OS-induced explants with or without 0.67 µg/mL LG. (**B**) Quantitative analysis of the optical density (OD) of the immunoreactive bands. Each column represents mean ± SD. * *p* < 0.05 vs. Ctrl; ** *p* < 0.01 vs. Ctrl; ^§^ *p* < 0.05 vs. OS; *n* = 3. Power value: 0.99. (**C**–**F**) Representative immunofluorescence images showing the presence of active caspase-3 immunolabeled cells in the inner nuclear layer (INL) and in the ganglion cell layer (GCL) in coronal sections of control explants (**C**), explants treated with 0.67 µg/mL LG alone (**D**), OS-induced explants (**E**), OS-induced explants incubated with 0.67 µg/mL LG (**F**). IPL: inner plexiform layer. 4′,6-diamidino-2-phenylindole (DAPI) counterstain. Calibration bar, 50 µm. (**G**): Quantitative analysis of the number of immunostained cells. Each column represents mean ± SD. ** *p* < 0.01 vs. Ctrl; ^§§^ *p* < 0.01 vs. OS; *n* = 4. Power value: 0.91.

Consistent with the qPCR data obtained with 0.67 μg/mL LG, OS was observed to induce a significant increase of active caspase-3 levels, while the administration of LG significantly decreased active caspase-3 in the presence of OS (Figure 2A,B). These Western blot data were confirmed by an immunofluorescence analysis of the localization of active caspase-3, which showed a significant increase of the number of immunolabeled profiles in the inner nuclear layer (INL) and in the ganglion cell layer (GCL) of OS-stressed explants and a complete recovery to control values in OS-stressed explants treated with LG (Figure 2C–G).

**Figure 3:**
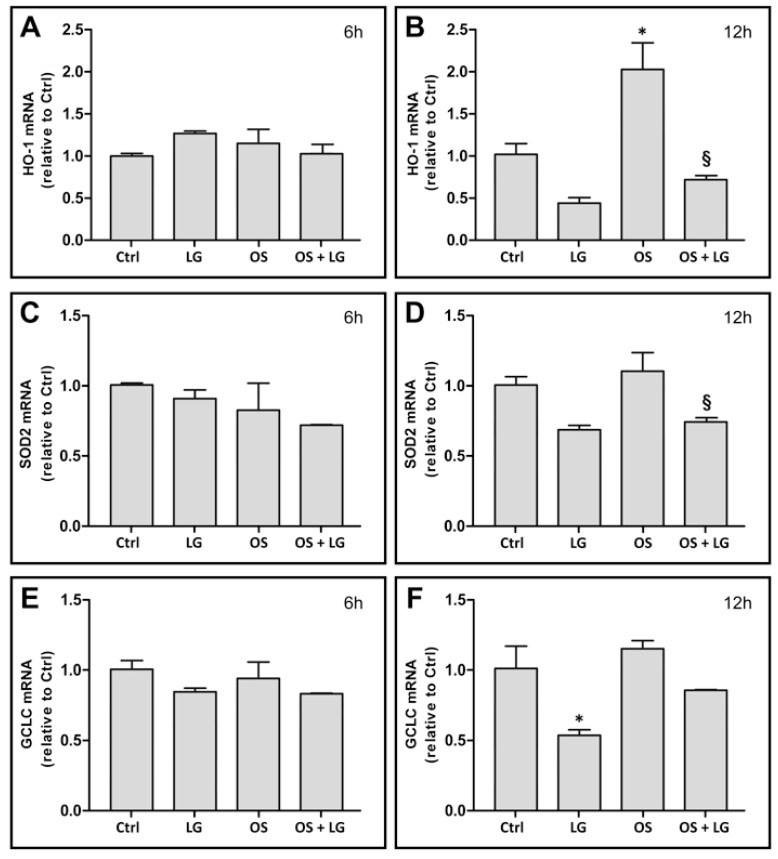
Heme oxygenase-1 (HO-1, (**A**,**B**)), superoxide dismutase 2 (SOD2, (**C**,**D**)), and glutamate-cysteine ligase catalytic subunit (GCLC, (**E**,**F**)) mRNA expression in retinal explants after 6 h (**A**,**C**,**E**) or 12 h (**B**,**D**,**F**) incubation. LG was administered at a concentration of 0.67 µg/mL. Each column represents mean ± SD. * *p* < 0.05 vs. Ctrl at 12 h; ^§^ *p* < 0.05 vs. OS at 12 h; *n* = 3 for all measures. Power value in (**B**,**D**,**F**) is 0.80.

To ascertain whether the effects of LG could be attributed to its antioxidant properties and to determine when LG treatment begins to be effective, the expression of the OS markers heme oxygenase-1 (HO-1), superoxide dismutase 2 (SOD2), and glutamate-cysteine ligase catalytic subunit (GCLC) was investigated after 6 or 12 h of incubation with or without LG. No significant effects of either OS or LG were observed after 6 h of incubation (Figure 3A,C,E). After 12 h, an increase of HO-1 mRNA levels was observed in OS-stressed explants (Figure 3B), while no effects of OS were recorded on the expression of either SOD2 (Figure 3D) or GCLC (Figure 3F) mRNA. In addition, OS-stressed explants treated with LG showed significant reduction of HO-1 (Figure 3B) and of SOD2 (Figure 3D) mRNA, while reduction of GCLC mRNA did not reach statistical significance (Figure 3F). These data indicate that, starting between 6 and 12 h from OS induction, the expression of antioxidant enzymes tends to increase, while LG treatment tends to reduce this increase. Interestingly, LG treatment also reduced the mRNA levels of OS markers in control explants, although a statistically significant decrease was observed only for GCLC mRNA (Figure 3F).

### 3.2. LG Treatment Does Not Affect Glycaemia and Body Weight of Diabetic Rats

**Figure 4:**
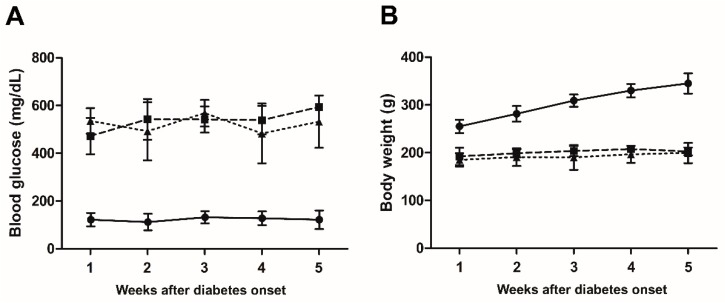
Blood glucose levels and body weight (means ± SD) of rats during five weeks after diabetes onset in STZ rats. Mean blood glucose levels (**A**) and body weight (**B**) were recorded in control rats (*n* = 5, circles), in STZ rats (*n* = 5, squares), and in STZ rats treated with LG (*n* = 5, triangles).

Normal blood glucose levels in control rats ranged between 108.5 and 147.0 mg/dL during the 5 weeks of the experiment (Figure 4A). They were about four times higher both in STZ rats and in STZ rats treated with LG (472.2–593.6 and 425.8–513.3 mg/dL, respectively). No significant differences in blood glucose levels were observed between STZ rats and STZ rats treated with LG.

The body weight (Figure 4B) ranged between 255.0 g and 345.0 g in control rats and it was consistently lower both in STZ rats and in STZ rats treated with LG (192.2–203.4 and 184.8–200.0 g, respectively). No significant differences in body weight were observed between STZ rats and STZ rats treated with LG.

### 3.3. LG Treatment Improves the Electroretinographic Responses of Diabetic Rats

**Figure 5:**
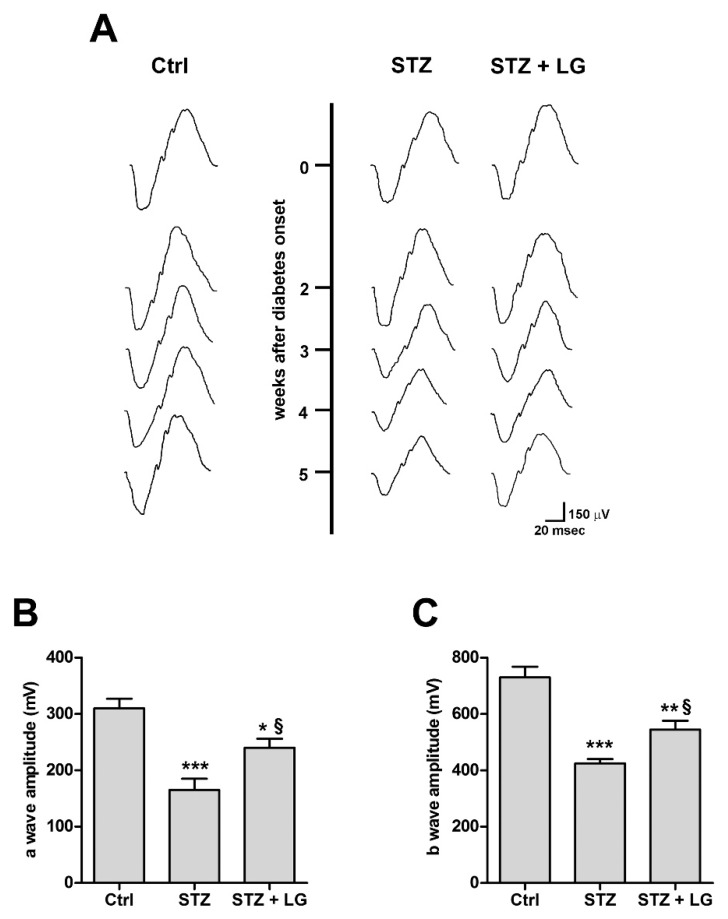
LG partially restored electroretinogram (ERG) responses. (**A**) Representative ERG waveforms in control and STZ or STZ + LG rats recorded at light intensity of 1 log cd-s·m^−2^ from 2 to 5 weeks after diabetes onset. (**B**,**C**) Means ± SEM of a- and b-wave amplitudes, respectively, in control rats, STZ rats, and STZ rats treated with LG 5 weeks after diabetes onset. Each column represents mean ± SD. * *p* < 0.05 vs. Ctrl; ** *p* < 0.01 vs. Ctrl; *** *p* < 0.001 vs. Ctrl; ^§^ *p* < 0.05 vs. STZ. *n* = 5 for all measures. Power value in (**B**,**C**) is 0.99.

To determine the effect of daily LG treatment on visual function in diabetic rats, we used full-field flash ERG and the results are reported in Figure 5. Consistent with previous observations [17], after 2 weeks from diabetes onset, the a- and b-waves of both STZ and STZ + LG rats were similar to those of control rats. Beginning at 3 weeks of diabetes, the a- and b-waves of both STZ and STZ + LG rats were decreased with respect to controls and there were no differences between the two groups. At week 4, the decrease of wave amplitude was more marked in the STZ than in the STZ + LG group, and statistically significant differences were recorded between the two groups. At week 5, the picture was the same as that at week 4, with the same differences in wave amplitudes between the two groups. These findings suggest that LG treatment protected both the inner and outer retina from the functional deficits typically induced by diabetes.

### 3.4. LG Treatment Reduces Retinal Apoptosis, VEGF Expression, and VEGFR2 Activation in Diabetic Rats

**Figure 6:**
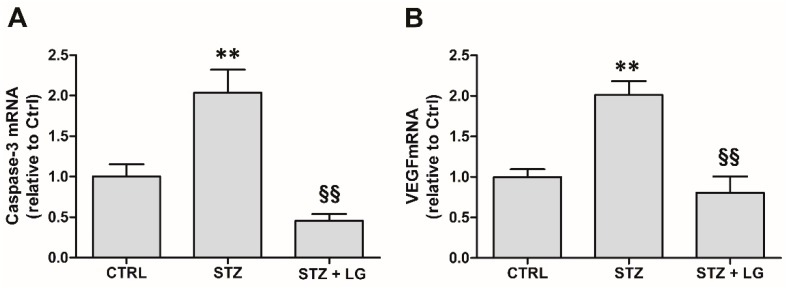
Caspase-3 (**A**) and VEGF (**B**) mRNA expressions evaluated with qPCR in the retinas of control rats and in retinas of STZ rats with or without LG treatment. ** *p* < 0.01 vs. Ctrl; ^§§^ *p* < 0.01 vs. STZ; *n* = 3 for all measures. Power value: 0.99 (**A**), 0.97 (**B**).

**Figure 7:**
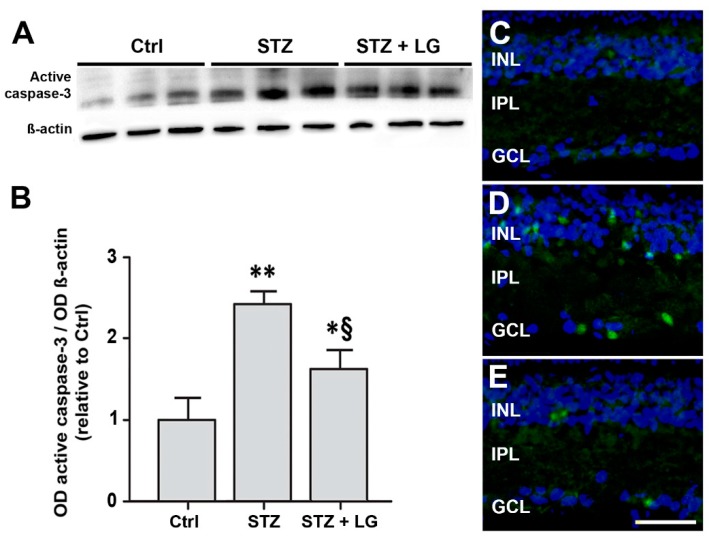
(**A**) Western blots showing immunoreactive bands of active caspase-3 and of ß-actin, used as an internal standard,** i**n control rat retinas and in retinas of STZ rats with or without LG treatment. (**B**) Quantitative analysis of the OD of the immunoreactive bands. Each column represents mean ± SD. * *p* < 0.05 vs. Ctrl; ** *p* < 0.01 vs. Ctrl; ^§^ *p* < 0.05 vs. STZ; *n* = 3 for all measures. Power value: 0.99. (**C**–**E**) Representative immunofluorescence images showing caspase-3 immunopositive cells in retinas of control rats (**C**), STZ rats (**D**), and STZ rats treated with LG (**E**). DAPI counterstain. Scale bar, 50 µm.

**Figure 8:**
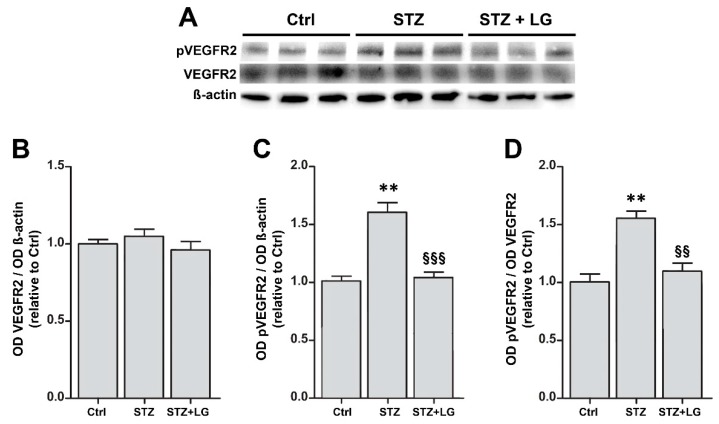
(**A**) Western blots showing immunoreactive bands of VEGF receptor 2 (VEGFR2), of phosphoVEGFR2 (pVEGFR2) and of ß-actin, used as an internal standard,** i**n control rat retinas and in retinas of STZ rats with or without LG treatment. (**B,C,D**) Graphs representing the ODs of the immunoreactive bands to quantify the protein expression of VEGFR2 (**B**), that of pVEGFR2 (**C**), and the ratio pVEGFR2/VEGFR2 as a measure of VEGFR2 activation (**D**). Each column represents mean ± SD. ** *p* < 0.01 vs. Ctrl; ^§§^ *p* < 0.01 vs. STZ; ^§§§^ *p* < 0.001 vs. STZ; *n* = 3 for all measures. Power value: 0.99.

Consistent with the findings in mouse retinal explants, in the retinas of STZ rats the expression of both caspase-3 and VEGF mRNAs was dramatically increased, while the treatment with LG completely inhibited these increases (Figure 6). Similarly, the levels of active caspase-3 were increased in retinas of STZ rats and were significantly reduced by LG treatment (Figure 7A,B). Active caspase-3 immunolabeled profiles, which were virtually absent in control retinas (Figure 7C), were numerous in the INL and in the GCL of STZ retinas (Figure 7D), while they were drastically decreased in the retinas of the STZ + LG group (Figure 7E). The changes of VEGF mRNA corresponded to changes in VEGFR2 activation. Indeed, as depicted in Figure 8, the diabetic condition determined a significant increase of VEGFR2 phosphorylation, while this increase was inhibited by LG treatment.

### 3.5. LG Treatment Protects the BRB in Diabetic Rats

**Figure 9:**
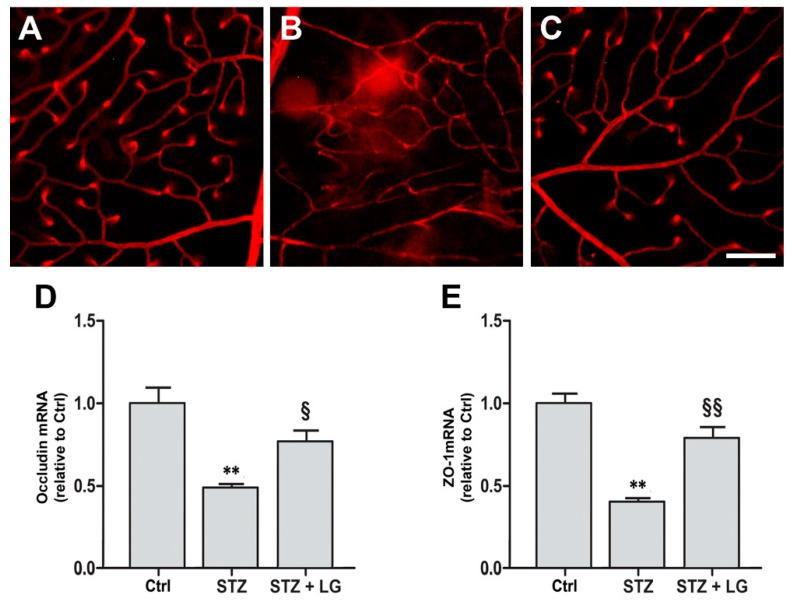
Effects of LG on blood-retinal barrier (BRB) integrity. Blood-retinal vascular leakage was evaluated with the Evans blue method in control rats (**A**), in STZ rats (**B**), and in STZ rats treated with LG (**C**). Scale bar: 100 μm. (**D**,**E**) Occludin and zonula occludens-1 (ZO-1), respectively, mRNA expression evaluated with qPCR in control retinas and in retinas of STZ rats with or without LG treatment. Each column represents mean ± SD. ** *p* < 0.01 vs. Ctrl; ^§^ *p* < 0.05 vs. STZ; ^§§^ *p* < 0.01 vs. STZ; *n* = 3 for all measures. Power value: 0.83 (**D**), 0.99 (**E**).

The whole retinal vasculature was visualized with Evans blue in control (Figure 9A), STZ (Figure 9B) and STZ + LG (Figure 9C) rats. Retinal vascular leakage was evident in STZ retinas and it was observed in different retinal regions. In retinas of the STZ + LG group, almost no vascular leakage could be detected and the retinal vessels were similar to those in control retinas. Consistent with these observations, the mRNA levels of occludin and of zonula occludens-1 (ZO-1), two proteins of the BRB, were significantly decreased in the retinas of diabetic rats, while they were similar to control levels in the retinas of rats treated with LG (Figure 9D).

### 3.6. LG Treatment Modifies Nrf2 Immunostaining Patterns in the Retina of Diabetic Rats

**Figure 10:**
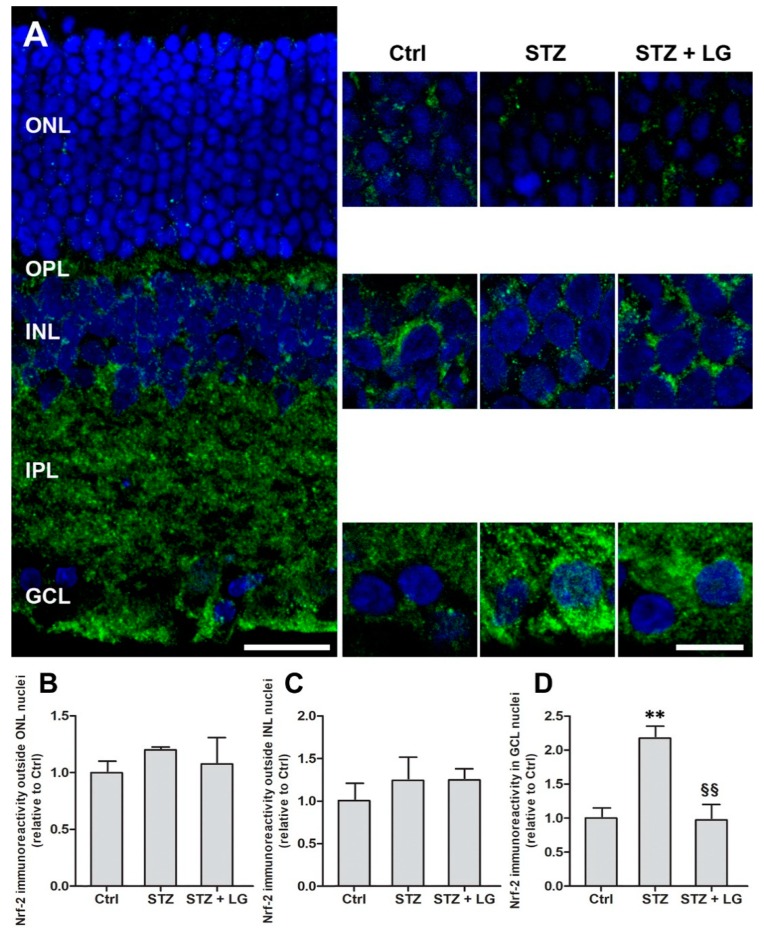
(**A**) Nuclear factor erythroid 2-related factor 2 (Nrf2) immunostaining patterns observed with the ApoTome microscope in coronal retinal sections of control rats, STZ rats, and STZ rats treated with LG. Left panel: low power extended focus image of a retinal section of a control rat showing extensive Nrf2 immunolabeling throughout the retinal layers. Scale bar, 20 μm. Right panels: higher power images of single focal planes showing changes in Nrf2 immunostaining in the ONL, INL, and GCL in the three experimental conditions. Scale bar, 10 μm. (**B**–**D**) Quantitative analysis of Nrf2 immunofluorescence levels in non-nuclear regions of the ONL (**B**) and of the INL (**C**) and within cell nuclei in the GCL (**D**). Each column represents mean ± SD. ** *p* < 0.01 vs. Ctrl; ^§§^ *p* < 0.01 vs. STZ; *n* = 4 for all measures. Power value: 0.94.

Nrf2 is a redox-sensitive transcription factor that, in response to increased free radicals, enters the cell nucleus and binds to the antioxidant response element, which initiates the transcription of antioxidant genes [18]. In our study, Nrf2 localization was evaluated with immunofluorescence to infer about the OS state of the retina. Nrf2 immunostaining patterns displayed some changes in the retinas of STZ rats with respect to controls, while they were similar to controls in the retinas of the STZ + LG group. As depicted in Figure 10A, Nrf2 immunostaining was present in all retinal layers. In particular, in the outer nuclear layer (ONL), in the INL, and in the GCL of control retinas, Nrf2 immunofluorescence was detected mostly around DAPI-stained cell nuclei, indicating a cytoplasmic localization. In the retinas of the STZ group, Nrf2 immunofluorescence seemed to be reduced in both the ONL and INL (Figure 10A), however, the quantitative analysis of immunofluorescence levels in non-nuclear regions of these two layers did not detect significant differences (Figure 10B,C). In contrast, in the GCL of STZ retinas, Nrf2 immunoreactivity was apparently distributed both around and within large DAPI-stained nuclei presumably belonging to ganglion cells. A quantitative evaluation indicated a significant increase of the levels of Nrf2 immunofluorescence within the nuclei of these cells in STZ rats, while the values recorded in the STZ + LG group were similar to control values (Figure 10D). These observations are consistent with a diabetes-induced Nrf2 nuclear translocation in ganglion cells and with an effect of LG against this phenomenon.

### 3.7. LG Treatment Reduces Glial Activation and Inflammation in the Retina of Diabetic Rats

**Figure 11:**
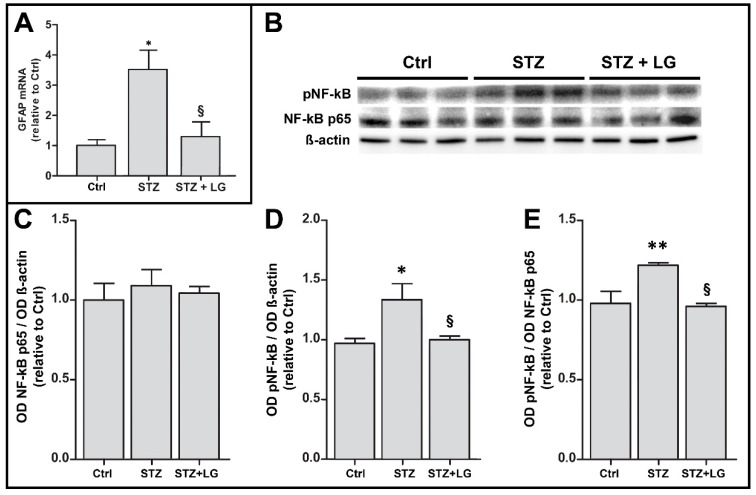
Effects of LG on inflammatory markers. (**A**) GFAP mRNA expression evaluated with qPCR in control retinas and in retinas of STZ rats with or without LG treatment. (**B**) Western blots showing immunoreactive bands of NF-kB, of pNF-kB and of ß-actin, used as an internal standard,** i**n control rat retinas and in retinas of STZ rats with or without LG treatment. (**C,D,E**) Graphs representing the ODs of the immunoreactive bands to quantify the protein expression of NF-kB (**C**), that of pNF-kB (**D**), and the ratio pNF-kB /NF-kB as a measure of NF-kB activation (**E**). Each column represents mean ± SD. * *p* < 0.05 vs. Ctrl; ** *p* < 0.01 vs. Ctrl; ^§^ *p* < 0.05 vs. STZ; *n* = 3 for all measures. Power value: 0.80 (**A**), 0.92 (**B**).

The inflammatory response was evaluated by assessing glial activation with measurements of glial fibrillary acidic protein (GFAP) mRNA expression and by determining the phosphorylation level of NF-kB with Western blot. GFAP mRNA expression was significantly increased in retinas of the STZ group with respect to controls, while in the STZ + LG group the levels of GFAP mRNA were similar to those in control retinas (Figure 11A). Similarly, the phosphorylation of NF-kB was significantly increased in the diabetic retinas and it was decreased to control values in the retinas of diabetic rats treated with LG (Figure 11B).

## 4. Discussion

In the present work, we hypothesized that LG, a substance of natural origin that is commercialized as a nutritional supplement, may be an interesting nutraceutical for use in DR treatments. Using an ex vivo model of the mouse retina, we observed that LG may play direct protective effects in the retina in the presence of OS. These findings were preliminary to investigations using the STZ rat model of experimental diabetes, where we found that orally administered LG exerts extensive protective effects both protecting retinal cells from apoptosis and limiting the extent of vascular lesions. Together, these data clearly demonstrate that LG exerts powerful protective effects in retinas suffering from stresses similar to those characterizing DR and that these effects are likely to be due to important antioxidant and anti-inflammatory properties of LG. LG exerts its protective actions independent from glycemic control and body weight, similar to other nutraceuticals with documented beneficial effects in rodent models of DR [19,20,21,22].

### 4.1. LG-Induced Neuroprotection Reduces Vascular Damage

In ex vivo mouse retinal explants exposed to OS as well as in retinas of STZ rats, LG reduces both apoptosis and VEGF overexpression. These findings are consistent with the view that in the stressed retina VEGF would be expressed and released to protect the retina by virtue of its documented neuroprotective properties [14,23,24,25,26]; however, in the presence of a protective factor such as LG, neuronal stress would be reduced and so would VEGF expression and release. In addition, as it could be expected, the reduced VEGF levels in the presence of LG also result in reduction of VEGFR2 activation. Consequently, the observed effects of LG leading to reduced vascular leakage and preservation of BRB protein expression are likely to be due to decreased VEGF expression and VEGFR2 activation. Indeed, VEGF upregulation is known to cause BRB breakdown inducing downregulation and phosphorylation of tight junction molecules such as occludin and ZO-1 [27,28,29]. Together, these data indicate that a neuroprotective strategy may positively impact on retinal vessels, decreasing BRB damage and, likely, other vascular lesions, in support of the idea that DR could be classified as a neurodegenerative retinal disease and that vascular defects are secondary to neuronal impairment [30,31].

### 4.2. LG Antiapoptotic Effects Result in Functional Recovery

LG reduces apoptosis in both the ex vivo retinal explants and the STZ rat model. In particular, LG treatment significantly reduces the increases in caspase-3 mRNA expression, active caspase-3 levels, and number of active caspase-3 immunolabeled cells provoked by OS or by diabetes. Antiapoptotic effects of LG have been documented in cultured human endothelial progenitor cells challenged with lipopolysaccharides [12]. Inhibition of retinal neuronal death results in ameliorated retinal function, as evaluated with ERG. However, functional recovery is not complete, as LG treated STZ rats display an ERG in which both a and b wave amplitudes are reduced with respect to control rats. The observation that there were no differences between the ERG responses at 4 and at 5 weeks suggests that further improvements of ERG responses in the STZ rats are unlikely. Similar observations have been reported for other substances of natural origin. Indeed, in the last few years a variety of nutraceuticals have been found to exert important antioxidant effects resulting in amelioration of ERG responses in different retinal disease or retinal injury models [32,33,34,35,36,37,38]. 

### 4.3. Antioxidant and Anti-Inflammatory Effects of LG

Our results show that both OS and inflammation are reduced by LG in an in vivo experimental model of DR. Antioxidant effects of LG are detected very early: our observations in ex vivo retinal explants suggest that OS reduction (indicated by decreased expression of antioxidant enzymes) is induced by LG just about the time when some effects of OS become visible (indicated by increased expression of HO-1 mRNA), that is after 12 h of incubation. In addition, LG effects are visible even in the absence of OS, since reduction of antioxidant enzyme expression is detected in control explants. This result is similar to data reported in a previous study [14] and can be explained recognizing that the preparation of the explant model (which includes retinal dissection and cutting) induces some damage and a certain level of OS even in control explants. 

It has been reported recently that activation of Nrf2 and consequent expression of antioxidant enzymes may protect the retina of STZ rats [39]. Our results show that both in the ex vivo explants and in the STZ rats, the antioxidant effects of LG are likely to be due to direct radical scavenging and reduction of OS, in line with previous observations reporting strong antioxidant activity of LG in an in vitro acellular system [8]. In these conditions, Nrf2 nuclear translocation and expression of antioxidant enzymes would be reduced, as suggested by our data indicating that LG treatment decreases the expression of these enzymes in OS-induced retinal explants and is likely to inhibit Nrf2 nuclear translocation in presumed ganglion cells of STZ-treated rats. These observations are different from those reported in rat hepatocytes, where Nrf2 nuclear translocation and induction of phase 2 enzymes were observed after LG treatment [6]. This discrepancy suggests that LG may express its antioxidant effects through different mechanisms in the central nervous system and in peripheral tissues. The effect of LG on NF-kB, instead, may rely on similar mechanisms in different cell types. Indeed, it is known that activation of the NF-kB pathway requires a number of phosphorylation steps to allow nuclear translocation of this transcription factor [40]; therefore, the reduction of NF-kB phosphorylation observed in the diabetic retina and the inhibition of nuclear translocation of NF-kB observed in hepatocytes [6] or in human endothelial progenitor cells [12] are likely to be parts of the same mechanism by which LG reduces the inflammatory response elicited in the presence of a stress. This action of LG also includes a regulation of GFAP mRNA expression, commonly used as a marker of reactive astrogliosis. Interestingly, NF-kB has been reported to play a key role in the transduction pathway leading to GFAP expression [41].

### 4.4. LG as an Appropriate Compound for the Treatment of DR

In general, it is clear that antioxidant treatments can reduce retinal damage and improve retinal function in many, if not all, retinal diseases. The choice of the appropriate compound depends on its availability, the possibility to use it as a dietary supplement, its antioxidant power, its efficient absorption in the intestine, and its capacity to cross the BRB and reach the retina in appropriate amounts. LG seems to possess these characteristics since it is available as a nutritional supplement and it is a very efficient antioxidant, thanks mainly to the fermentation step that enriches LG with polyphenols and alpha-lipoic acid [6]. Interestingly, polyphenol-rich nutraceuticals and alpha-lipoic acid have been demonstrated to be highly effective in reducing VEGF upregulation in DR models [21,42,43,44,45,46]. In addition, the reported increase of antioxidant activity in the plasma of subjects after LG intake [47], and the observation that the effects of LG in the retinas of STZ-treated rats are comparable to those detected in OS-induced retinal explants indicate that LG is efficiently absorbed in the intestine and reaches the retina in concentrations that are in the range of those that we have used in the ex vivo experiments.

## 5. Conclusions

The data reported in this study indicate that LG deserves attention as a promising nutraceutical that may help in the treatment of DR and, likely, of other retinal diseases. Of course, there are limitations including the lack of information about possible improvements of LG effects through the application of different preparation protocols and the difficulty of translating the present results into clinical practice. Therefore, further studies are needed to compare the efficacy of different formulations of LG and to better clarify mechanistic aspects of its antioxidant and anti-inflammatory effects.

## Figures and Tables

**Table 1:** Primer sequences.

	Gene	Forward Primer (5′-3′)	Reverse Primer (5′-3′)
**Mouse**	*Caspase-3*	GCACTGGAATGTCATCTCGCTCTG	GCCCATGAATGTCTCTCTGAGGTTG
	*GCLC*	GGGGTGACGAGGTGGAGTA	GTTGGGGTTTGTCCTCTCCC
	*HO-1*	AAGCCGAGAATGCTGAGTTCA	GCCGTGTAGATATGGTACAAGGA
	*Rpl13a*	CACTCTGGAGGAGAAACGGAAGG	GCAGGCATGAGGCAAACAGTC
	*SOD2*	CAGACCTGCCTTACGACTATGG	CTCGGTGGCGTTGAGATTGTT
	*VEGF*	GCACATAGGAGAGATGAGCTTCC	CTCCGCTCTGAACAAGGCT
**Rat**	*Caspase-3*	CCTTTCCTCTCCACCGTAGA	AGATGCCACCTCTCCTTTCC
	*GFAP*	TGACGCCTCCACTCCCTGCC	CATCTCCGCACGCTCGCTGG
	*Occludin*	TTTCATGCCTTGGGGATTGAG	GACTTCCCAGAGTGCAGAGT
	*Rpl13a*	GGATCCCTCCACCCTATGACA	CTGGTACTTCCACCCGACCTC
	*VEGF*	TGTGAGCCTTGTTCAGAGCGG	ACTCAAGCTGCCTCGCCTTGC
	*ZO-1*	AGTCTCGGAAAAGTGCCAGG	GGGCACCATACCAACCATCA

## Supplementary Materials

The following are available online at http://www.mdpi.com/2072-6643/10/12/1932/s1, Figure S1: Dose-response of Lisosan G (LG) effects on the expression of Caspase 3 mRNA (**A**) and of VEGF mRNA (**B**) in mouse retinal explants exposed to oxidative stress (OS).
