# Supplementary material for: Lisosan G Protects the Retina from Neurovascular Damage in Experimental Diabetic Retinopathy
Source: Nutrients. 2018 Dec 5;10(12):1932. doi: 10.3390/nu10121932 (PMC6316708; doi:10.3390/nu10121932)
Supplement: Supplementary file 1 [file nutrients-10-01932-s001.pdf]

Supplementary Materials

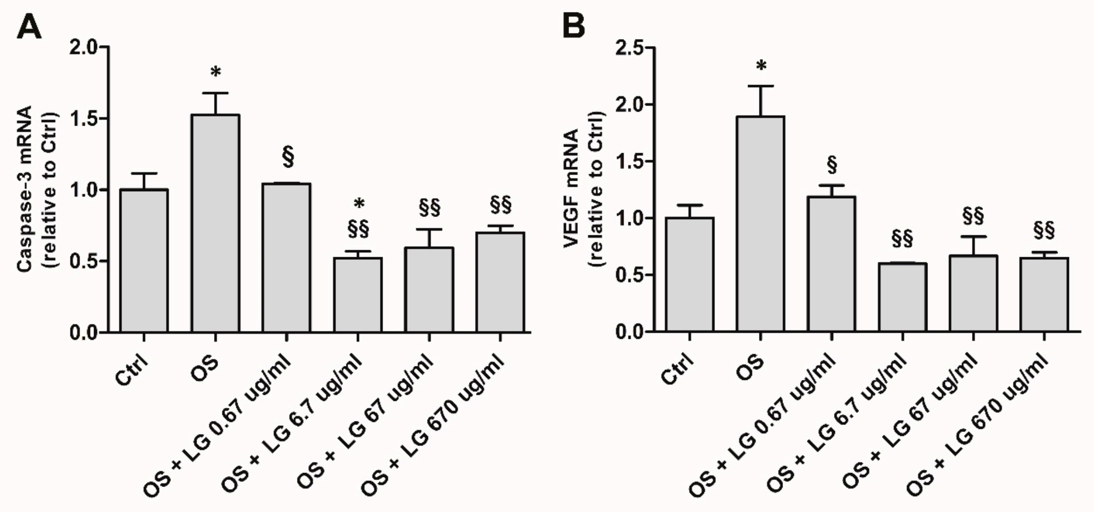

Figure S1. Dose-response of Lisosan G (LG) effects on the expression of Caspase 3 mRNA (**A**) and of VEGF mRNA (**B**) in mouse retinal explants exposed to oxidative stress (OS). A dose-dependent effect of LG was observed with doses of 0.67 and 6.7 μg/ml. LG at higher doses did not induce any further changes in either caspase-3 or VEGF mRNA expression. Therefore, the experiments were conducted using 0.67 or 6.7 μg/ml LG. Each column represents mean ± SD. *$p<0.05$ vs Ctrl; [§]$p<0.05$ vs OS; [§§]$p<0.01$ vs OS; n = 3.
